# Salivary Antioxidant Barrier, Redox Status, and Oxidative Damage to Proteins and Lipids in Healthy Children, Adults, and the Elderly

**DOI:** 10.1155/2019/4393460

**Published:** 2019-12-05

**Authors:** Mateusz Maciejczyk, Anna Zalewska, Jerzy Robert Ładny

**Affiliations:** ^1^Department of Hygiene, Epidemiology and Ergonomics, Medical University of Bialystok, Bialystok, Poland; ^2^Experimental Dentistry Laboratory, Medical University of Bialystok, Bialystok, Poland; ^3^Department of Emergency Medicine and Disaster, Medical University Bialystok, Bialystok, Poland

## Abstract

Despite the proven role of oxidative stress in numerous systemic diseases and in the process of aging, little is still known about the salivary redox balance of healthy children, adults, and the elderly. Our study was the first to assess the antioxidant barrier, redox status, and oxidative damage in nonstimulated (NWS) and stimulated (SWS) saliva as well as blood samples of healthy individuals at different ages. We divided 90 generally healthy people into three equally numbered groups based on age: 2–14 (children and adolescents), 25–45 (adults), and 65–85 (elderly people). Antioxidant enzymes (salivary peroxidase (Px), glutathione peroxidase (GPx), catalase (CAT), and superoxide dismutase-1 (SOD)), nonenzymatic antioxidants (reduced glutathione (GSH) and uric acid (UA)), redox status (total antioxidant capacity (TAC), total oxidant status (TOS), and oxidative stress index (OSI)), and oxidative damage products (advanced glycation end products (AGE), advanced oxidation protein products (AOPP), and malondialdehyde (MDA)) were evaluated in NWS and SWS as well as in erythrocyte/plasma samples. We demonstrated that salivary and blood antioxidant defense is most effective in people aged 25–45. In the elderly, we observed a progressive decrease in the efficiency of central antioxidant systems (↓GPx, ↓SOD, ↓GSH, and ↓TAC in erythrocytes and plasma vs. adults) as well as in NWS (↓Px, ↓UA, and ↓TAC vs. adults) and SWS (↓TAC vs. adults). Both local and systemic antioxidant systems were less efficient in children and adolescents than in the group of middle-aged people, which indicates age-related immaturity of antioxidant mechanisms. Oxidative damage to proteins (↑AGE, ↑AOPP) and lipids (↑MDA) was significantly higher in saliva and plasma of elderly people in comparison with adults and children/adolescents. Of all the evaluated biomarkers, only salivary oxidative damage products generally reflected their content in blood plasma. The level of salivary redox biomarkers did not vary based on gender.

## 1. Introduction

The oral cavity is the only place in the body that is directly exposed to numerous environmental factors, such as food, alcohol, cigarette smoke, medicines, air pollution, and pathogenic microorganisms. These factors have been demonstrated to act prooxidatively by producing reactive oxygen (ROS) and nitrogen (RNS) species [1, 2]. Excessive activity of ROS and RNS results in oxidative/nitrosative damage to proteins, lipids, and DNA, which leads to alternations in cellular metabolism and is referred to as oxidative stress [3]. No wonder that saliva produced by salivary glands is a rich source of antioxidants protecting us against disturbances of redox homeostasis not only in the oral cavity but also in the entire body [1, 4]. Antioxidants contained in saliva include antioxidant enzymes (salivary peroxidase (Px), catalase (CAT), peroxidase (SOD), and glutathione reductase (GR)) as well as nonenzymatic antioxidants (uric acid (UA), reduced glutathione (GSH), albumin, and lactoferrin) and polyphenols [1, 4]. Thus, the oral cavity forms the first line of defense against oxidative stress [1, 2]. However, overproduction of ROS occurs not only under the influence of environmental factors but also as a result of the aging of the body and systemic diseases [1, 2, 4].

Saliva is a secretion produced by three pairs of large salivary glands (parotid, submandibular, and sublingual glands) as well as numerous smaller glands located on the lip, tongue, and cheek mucosa. Saliva consists of 99% water, and the remaining percentage is comprised of inorganic (sodium, potassium, chlorides, and phosphates) and organic (mucins, immunoglobulins, *α*-amylase, lipids, and antioxidants) components [5, 6]. Generally, the rate of saliva production depends on the degree of autonomic nervous system excitation, and its composition and volume also vary based on age and gender [7, 8]. It has been demonstrated that saliva secretion decreases with age, is lower in women, and contains larger amounts of sodium, calcium, and phosphorus in men [8, 9]. Previously, we also showed disturbances in the antioxidant barrier and increased oxidative damage in saliva and blood of elderly patients with dementia [10, 11]. However, little is still known about the influence of gender and age on the oxidative/reductive balance of saliva and the entire body of healthy individuals. Although oxidative stress plays a key role in the process of aging, no studies have been conducted to compare salivary redox homeostasis in young and elderly healthy people. What is more, there have been no studies comparing the biochemical composition of nonstimulated saliva (NWS), stimulated saliva (SWS), and blood in healthy subjects of different ages. Saliva is used more and more often as a diagnostic material [12–14]. Therefore, it is advisable to assess the correlation of salivary oxidative stress biomarkers and their level in blood plasma. Bearing this in mind, our study is aimed at evaluating enzymatic and nonenzymatic antioxidant barriers, redox status, and oxidative damage to proteins and lipids in NWS, SWS, and blood plasma/erythrocytes in healthy children, adults, and elderly people.

## 2. Materials and Methods

The study was approved by the Bioethics Committee of the Medical University of Bialystok, Poland (permission numbers R-I-002/62/2016 and R-I-002/43/2018). All participants (or their legal guardians) consented in writing to participate in the experiment. For the detailed experimental protocol, we followed the methods of our previous study [11].

### 2.1. Subjects

90 patients of the Specialist Dental Clinic (Department of Restorative Dentistry) of the Medical University of Bialystok were classified for the study. The experiment included generally healthy individuals with body mass index (BMI) between 18.5 and 24.5, who have never suffered from periodontitis, gingivitis, and cancer, as well as metabolic (e.g., type 1 diabetes and obesity), cardiovascular (e.g., arrhythmias and conductivity disorders), neuropsychiatric (e.g., Alzheimer's disease, Parkinson's disease, and dementia), kidney, liver, thyroid, lung, and gastrointestinal diseases. Additionally, the study excluded subjects with autoimmune (e.g., rheumatoid arthritis, scleroderma, and Sjögren's syndrome), infectious (e.g., infection with human immunodeficiency virus (HIV) and hepatitis C virus (HCV)), and gastrointestinal disorders, as well as smokers, alcohol-dependent subjects, and pregnant women. All subjects had normal blood count results (erythrocytes, hemoglobin, hematocrit, leukocytes, and platelets) and normal biochemical blood results (CRP, sodium, potassium, creatinine, and ALT), and had not taken antibiotics, hormones, nonsteroidal anti-inflammatory drugs (NSAIDs), dietary supplements, and vitamins for the last 3 months.

All participants of the study were divided into three groups based on age: 2–14 (children and adolescents), 25–45 (adults), and 65–85 (elderly people). Each group consisted of 30 subjects: 15 men and 15 women. The division into age groups was developed based on the WHO classification, considering the most common intervals in the standard population distribution. Additionally, at such age ranges, the Ministry of Health in Poland, as well as the Polish Stomatological Society, carries out epidemiological studies of oral health. The number of subjects was determined based on our previous experiment, assuming that the power of the test would equal 0.9.

Clinical data of subjects are presented in Table 1.

### 2.2. Saliva Collection

The studied material was mixed saliva (both NWS and SWS) collected via the spitting method after 2-hour abstinence from solid and liquid food (other than mineral water) as well as oral hygiene procedures. All subjects had refrained from taking any medications for 8 hours before the sampling. Saliva collection took place in a separate, cozy room, in a sitting position, with the head slightly bent downwards. To minimize the impact of daily changes on salivary secretion, saliva was collected between 7 am and 9 am upon 5-minute adaptation to the environment. After this time, each subject rinsed the mount three times with distilled water. Saliva was collected into a sterile Falcon tube placed in an ice container. Saliva collected within the first minute was discarded [15]. NWS was collected in the amount of up to 5 mL for no more than 15 minutes [11]. Saliva secretion was stimulated by dropping 10 *μ*L of 2% citric acid on the center of the tongue every 30 seconds. SWS was collected in the same manner as NWS, but for 5 min [11]. The volume of each sample was measured by an automatic pipette calibrated to 0.1 mL. Immediately after collection, the saliva was centrifuged (20 minutes, +4°C, 5000 × g; MPW 351, MPW Med. Instruments, Warsaw, Poland) and frozen at -80°C until assayed. The supernatant was preserved for further research. In order to protect the samples against oxidation, an antioxidant was added (10 *μ*L of 0.5 M butylated hydroxytoluene for 1 mL of salivary supernatant) [15]. The salivary flow was calculated by dividing the volume of saliva by the time necessary to collect it, and expressed in mL/min.

### 2.3. Dental Examination

Dental examination was performed in artificial light (10,000 lx) according to the World Health Organization criteria [16]. The examination included DMFT (decayed, missing, filled teeth), PBI (Papilla Bleeding Index), and GI (Gingival Index). The DMFT index is the sum of teeth with caries (D), teeth extracted because of caries (M), and teeth filled due to the occurrence of caries (F). The PBI expresses the intensity of bleeding from the gingival papilla after probing, while GI indicates qualitative changes in the gingivae. In children and adolescents, dmft for primary teeth was also assessed [16]. The clinical examination was performed by the same dentist after NWS and SWS collection. In 40 subjects, the interrater reliability, i.e., agreements among the examiner and two other experienced dentists, was assessed. The reliability for DMFT was *r* = 0.96; for PBI: *r* = 0.98; and for GI: *r* = 0.96.

### 2.4. Blood Collection

10 mL of venous blood was collected using the S-Monovette® K3 EDTA blood collection system (Sarstedt). All samples were taken on an empty stomach after an overnight rest. Blood was centrifuged (10 min, +4°C, 1500 × g), and plasma (upper layer after centrifugation) was collected immediately. Erythrocytes (bottom layer) were rinsed three times with cold saline (0.9% NaCl) and then hemolyzed by adding 9 volumes of cold 50 mM phosphate buffer, pH 7.4 (1 : 9, *v*/*v*) [11, 15]. No hemolysis was observed in any of the collected samples. Similarly to NWS and SWS, an antioxidant (10 *μ*L of 0.5 M butylated hydroxytoluene for 1 mL of blood) was added [11, 15]. Samples were frozen at -80°C until assayed.

### 2.5. Redox Analysis

The performed analysis included the determination of enzymatic antioxidants (salivary peroxidase (Px, EC 1.11.1.7), glutathione peroxidase (GPx, EC 1.11.1.9), catalase (CAT, EC 1.11.1.6), and superoxide dismutase-1 (SOD, EC 1.15.1.1)), nonenzymatic antioxidants (reduced glutathione (GSH) and uric acid (UA)), and redox status (total antioxidant capacity (TAC), total oxidant status (TOS), and oxidative stress index (OSI)), as well as oxidative damage products (advanced glycation end products (AGE), advanced oxidation protein products (AOPP), and malondialdehyde (MDA)).

Nonenzymatic antioxidants and oxidative damage products were assayed in NWS, SWS, and plasma samples, while antioxidant enzymes were assayed in NWS, SWS, and erythrocytes [11, 15]. Unless stated otherwise, all reagents were purchased from Sigma-Aldrich (Poland, Germany, or USA). On the day of the biochemical tests, the material was slowly thawed at 4°C. All assays were performed in duplicate samples and standardized to 1 mg of the total protein. The absorbance/fluorescence was measured using a 96-well microplate reader (Infinite M200 PRO Multimode, Tecan).

### 2.6. Antioxidant Assays

Px activity was assessed colorimetrically based on the reduction of 5,5′-dithiobis-(2-nitrobenzoic acid) (DTNB) to thionitrobenzoic acid [17]. A decrease in the absorbance of thionitrobenzoic acid was measured at 412 nm wavelength. The activity of erythrocyte GPx was determined colorimetrically at 340 nm based on the reduction of organic peroxides in the presence of NADPH (reduced nicotinamide adenine dinucleotide phosphate) [18]. One unit of GPx activity was assumed to catalyze the oxidation of 1 *μ*mol of NADPH per minute. CAT activity was assessed colorimetrically by measuring the decomposition rate of hydrogen peroxide in the sample at 240 nm wavelength [19]. One unit of CAT activity was defined as the amount of enzyme that decomposes 1 mmol hydrogen peroxide per minute. SOD activity was analyzed colorimetrically at 480 nm by measuring the inhibition rate of adrenaline oxidation to adrenochrome [20]. It was assumed that one unit of SOD activity inhibits the oxidation of adrenaline by 50%.

GSH content was assessed colorimetrically based on the reaction with DTNB [21]. The absorbance of the resulting complex was measured at 412 nm wavelength. UA concentration was determined with a kit supplied by BioAssay Systems (QuantiChrom™ Uric Acid Assay Kit DIUA-250; BioAssay Systems, Hayward, USA). This method used 2,4,6-tripyridyl-s-triazine, and absorbance of the resulting complex was measured at 490 nm wavelength.

### 2.7. Redox Status

TAC was analyzed colorimetrically at 660 nm based on the reaction with 2,2-azino-bis-3-ethylbenzothiazoline-6-sulfonic acid radical cation (ABTS^•+^) [22]. TAC level was calculated from the calibration curve for Trolox (6-hydroxy-2,5,7,8-tetramethylchroman-2-carboxylic acid). TOS was assessed bichromatically (560/800 nm) based on the oxidation of Fe^2+^ to Fe^3+^ in the presence of the oxidants contained in the sample. TOS level was calculated from the calibration curve for hydrogen peroxide and expressed as 1-micromolar hydrogen peroxide equivalent per mg protein. Oxidative stress index (OSI) was calculated by dividing TOS by TAC and expressed in % [15].

### 2.8. Oxidative Damage Assays

The content of AGE was estimated fluorimetrically by measuring AGE-specific fluorescence (350 nm/440 nm wavelength) in 96-well black-bottom microplates [23]. The concentration of AOPP was analyzed colorimetrically at 340 nm by measuring the oxidative capacity of iodine ion [23]. For AGE and AOPP determination, all samples were diluted 1 : 50 (*v*/*v*) in phosphate-buffered saline, pH 7.2.

Lipid peroxidation was estimated by measuring MDA by the thiobarbituric acid reactive substance (TBARS) method [24]. Absorbance was measured colorimetrically at 532 nm, and MDA concentration was calculated from the calibration curve for 1,3,3,3-tetraethoxypropane.

### 2.9. Total Protein Assay

Total protein content was measured using the bicinchoninic acid (BCA) method [25] with the commercial Pierce BCA Protein Assay Kit (Thermo Fisher Scientific, Rockford, IL, USA). Bovine serum albumin (BSA) was used as a standard.

### 2.10. Statistical Analyses

The statistical analysis was performed using GraphPad Prism 7 (GraphPad Software, La Jolla, CA, USA) and Microsoft Excel 16.16.10 for MacOS. Specific analyses included two-way ANOVA and post hoc Tukey's HSD (honestly significant difference) test, as well as ANOVA and Student's *t*-test. The associations between the variables were assessed using Pearson's correlation coefficient. The statistical significance was assumed as *p* < 0.05.

Importantly, in children aged 2-14 years, we did not observe any differences in the assessed parameters depending on the primary and deciduous teeth, and therefore all patients were classified into one group.

## 3. Results

### 3.1. Antioxidant Defense

**Figure 1:**
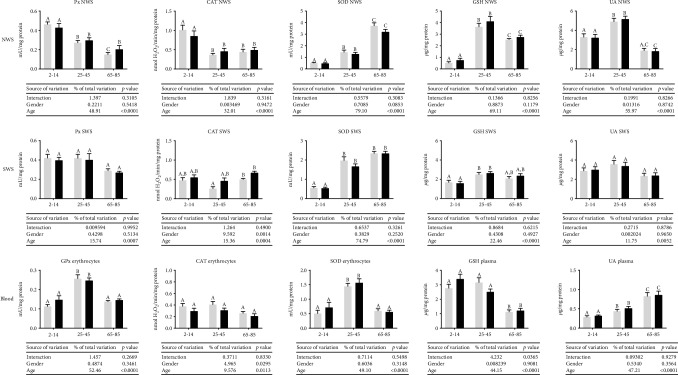
Enzymatic and nonenzymatic antioxidants in NWS, SWS, and blood plasma/erythrocytes of healthy children (aged 2-14), adults (aged 25-45), and elderly people (aged 65-85). Data are shown as mean ± SD. Grey bars represent men, while black represent women. Means without a common letter statistically differ (*p* < 0.05). CAT—catalase; GPx—glutathione peroxidase; GSH—reduced glutathione; NWS—nonstimulated whole saliva; Px—salivary peroxidase; SOD—superoxide dismutase-1; SWS—stimulated whole saliva; UA—uric acid.

The activity of Px in NWS was significantly higher in the group of children and adolescents (both in women and men) compared to middle-aged and elderly people, as well as in the group of 25-45-year-olds compared to participants aged 65–85 (except for women). CAT activity in NWS was significantly higher in the youngest subjects (aged 2–14) compared to the other groups. We also observed increased SOD activity in NWS of older people compared to the middle aged as well as children and adolescents. In NWS, the concentration of GSH was considerably higher in people aged 25–45 compared to those aged 2–14 and 65–85. Both in women and men, the concentration of UA in NWS was the highest in middle-aged people, and significantly lower in the elderly compared to children and adolescents. The results of a two-way analysis of variance (ANOVA) indicated that the NWS antioxidant barrier depends on age but is not related to gender or age-gender interaction (Figure 1).

In SWS, Px activity did not differ significantly between the individual groups of subjects, while CAT activity was considerably higher in women and men aged 65–85 compared to middle-aged men. SOD activity was significantly higher in the group of healthy people aged 25–45 and 45–65 than in children and adolescents. The concentration of GSH was significantly higher in the stimulated saliva of middle-aged people compared to children and adolescents (both in boys and girls), while the concentration of UA did not differ among all the study groups. The results of two-way ANOVA analysis revealed that the enzymatic and nonenzymatic antioxidant defense barrier of stimulated saliva depends on Px, SOD, GSH, and UA, and in the case of CAT, also on gender (Figure 1).

In both women and men, GPx and SOD activity in erythrocytes was significantly higher in middle-aged people compared to children, adolescents, and the elderly, whereas CAT activity did not statistically differ among all the study groups. The concentration of GSH was significantly reduced in the elderly compared to other groups. We also demonstrated increased UA concentration in the plasma of the elderly compared to people aged 25–45 and 2–14. The results of the two-way analysis of variance (ANOVA) indicated that central antioxidant defense depends mainly on the age of subjects (Px, SOD, GSH, and UA), and in the case of CAT, also on gender (Figure 1).

### 3.2. Redox Status

**Figure 2:**
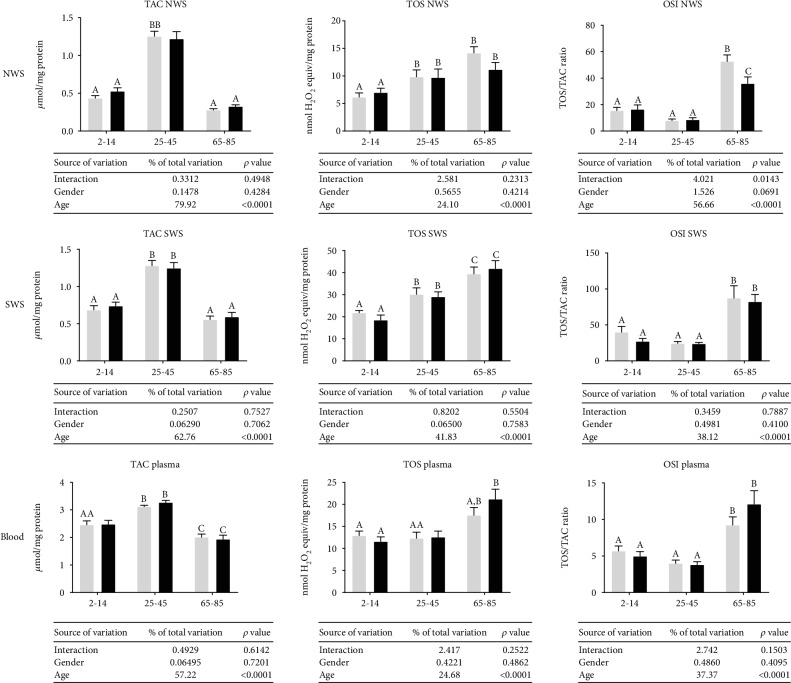
Redox status in NWS, SWS, and plasma healthy children (aged 2-14), adults (aged 25-45), and elderly people (aged 65-85). Data are shown as mean ± SD. Grey bars represent men, while black represent women. Means without a common letter statistically differ (*p* < 0.05). OSI—oxidative stress index; NWS—nonstimulated whole saliva; SWS—stimulated whole saliva; TAC—total antioxidant capacity; TOS—advanced oxidation protein products.

Generally, in NWS, SWS, and blood plasma alike, TAC was significantly higher in the group of middle-aged people compared to children and adolescents as well as the elderly. TOS and OSI were considerably higher in older people compared to those aged 2–14 and 25–45 (both in women and men). The results of the two-way ANOVA analysis indicated that the redox status depends only on age (Figure 2).

### 3.3. Oxidative Damage to Proteins and Lipids

**Figure 3:**
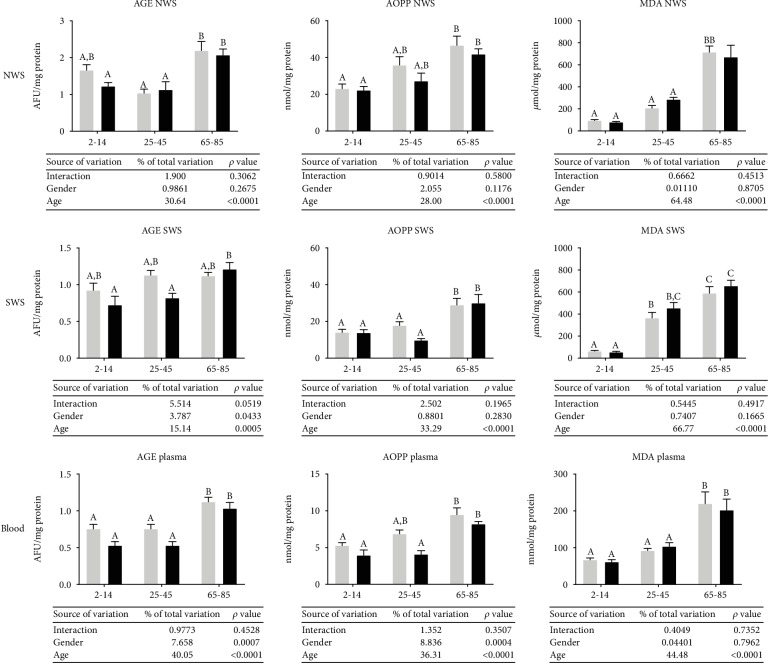
Oxidative damage in NWS, SWS, and plasma of healthy children (aged 2-14), adults (aged 25-45), and elderly people (aged 65-85). Data are shown as mean ± SD. Grey bars represent men, while black represent women. Means without a common letter statistically differ (*p* < 0.05). AGE—advanced glycation end products; AOPP—advanced oxidation protein products; MDA—malondialdehyde; NWS—nonstimulated whole saliva; SWS—stimulated whole saliva.

AGE and MDA levels were significantly higher in NWS of older people as compared to the middle-aged as well as children and adolescents. The AOPP concentration in the NWS of the elderly was considerably higher only compared to people aged 2–14. The results of the ANOVA showed that oxidative damage to proteins and lipids depends on age (Figure 3).

In SWS, AGE fluorescence was significantly higher in older women compared to girls aged 2–14 and women aged 25–45. The concentration of AOPP and MDA was considerably higher in the elderly compared to other groups (both in women and men). However, the results of the two-way ANOVA indicated that oxidative damage in SWS depends only on age, but is not connected with gender or age-gender interaction (Figure 3).

The levels of AGE, AOPP, and MDA in plasma were significantly higher in the elderly compared to the other groups. The results of the two-way ANOVA analysis revealed that oxidative damage to lipids depends on age (MDA), while oxidative damage to proteins (AGE and AOPP) depends on both age and gender (Figure 3).

### 3.4. Differences between NWS and SWS

In SWS of children aged 2-14, CAT activity and TAC levels were significantly higher, while AGE and MDA levels were statistically lower compared to NWS (Table 2).

In adults, Px and SOD activity as well as TOS and MDA levels were significantly higher in SWS, while GSH and UA levels and AOPP content were significantly lower compared to NWS (Table 2).

In the elderly aged 65-85, CAT and Px activity and TAC levels were significantly higher, while SOD activity and GSH and AGE levels were statistically lower in SWS compared to NWS (Table 2).

### 3.5. Correlations

**Figure 4:**
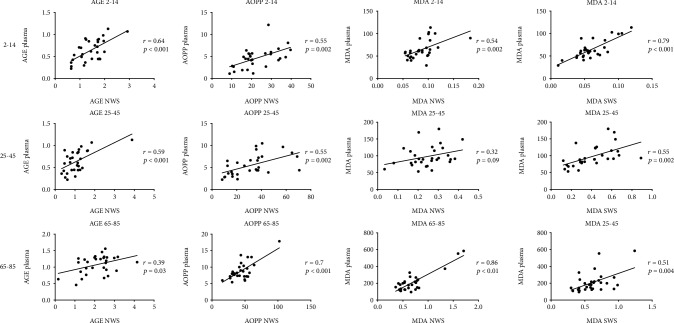
Correlations between salivary and plasma redox biomarkers in healthy children (aged 2-14), adults (aged 25-45), and elderly people (aged 65-85). AGE—advanced glycation end products; AOPP—advanced oxidation protein products; MDA—malondialdehyde; NWS—nonstimulated whole saliva; SWS—stimulated whole saliva.

The statistically significant correlations between salivary and plasma redox biomarkers are presented in Figure 4. Generally, we showed a positive correlation between the NWS content of AGE, AOPP, and MDA compared to plasma as well as MDA in SWS compared to plasma (Figure 4). However, there were no significant correlations between antioxidant concentration/activity in saliva and their level in blood plasma/erythrocytes (Tables [Supplementary-material supplementary-material-1] in the supplementary materials).

**Figure 5:**
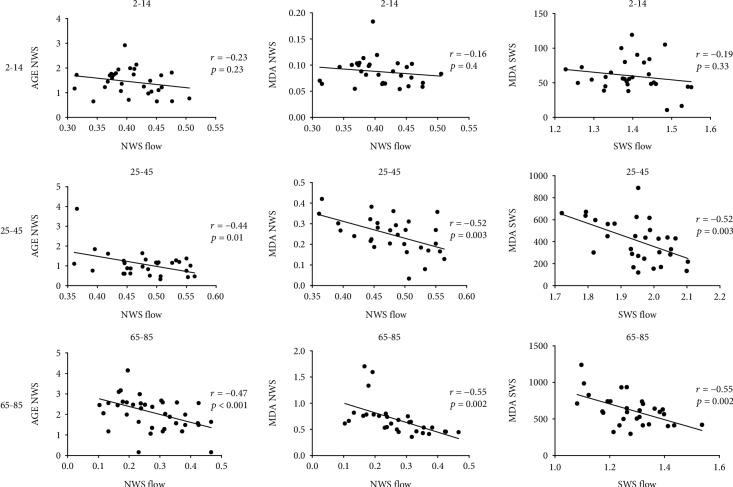
Correlations between salivary redox biomarkers and salivary flow rate in healthy children (aged 2-14), adults (aged 25-45), and elderly people (aged 65-85). AGE—advanced glycation end products; MDA—malondialdehyde; NWS—nonstimulated whole saliva; SWS—stimulated whole saliva.

In adults and the elderly, AGE fluorescence correlated negatively with the nonstimulated salivary secretion. Similarly, we recorded a negative correlation between salivary MDA concentration and the nonstimulated/stimulated salivary flow rate (Figure 5).

## 4. Discussion

Our research was the first to compare antioxidant defense, redox status, and oxidative damage to proteins and lipids in saliva and blood of healthy children, adults, and the elderly. In general, the antioxidant barrier of saliva is most effective in middle-aged people. It weakens with age and is accompanied by oxidative modifications of proteins and lipids as well as a decrease in saliva secretion. Although the concentration/activity of salivary antioxidants does not correlate with their levels in plasma and erythrocytes, the concentration of oxidative stress products in NWS reflects their content in the blood. Moreover, the level of salivary oxidative stress generally does not depend on gender.

Oxygen free radicals are involved in numerous biological processes, both in the healthy and ill bodies. As mediators and regulators, they influence gene expression, modulate cell differentiation and proliferation, and induce cell death through apoptosis [26, 27]. ROS also participate in the body's defense processes: neutralization of pathogenic organisms and detoxification of xenobiotics [27]. Under physiological conditions, increased production of free radicals is compensated by antioxidant systems, which prevents oxidative damage to cell components. However, long-term overproduction of ROS disturbs the redox balance of the body (in favor of oxidation reactions), leading to lipid peroxidation, cracking of DNA and RNA strands, and creation of cross-links in proteins. These processes play a key role in the pathogenesis of most diseases as well as in the aging process [27–29].

Although redox imbalances have been observed in the elderly, little is still known about the effectiveness of the antioxidant barrier and oxidative damage to proteins and lipids in healthy children and adults. We have demonstrated that antioxidant defense is most effective in people aged 25–45. In the elderly (65–85 years of age), we observed a progressive decrease in the efficiency of the antioxidant systems at the central level (↓GPx, ↓SOD, ↓GSH, ↓TAC, ↑TOS, and ↑OSI in erythrocytes and plasma vs. adults) as well as in NWS (↓Px, ↓UA, ↓TAC, and ↑OSI vs. adults) and SWS (↓TAC, ↑TOS, and ↑OSI vs. adults). However, the increased activity of salivary antioxidant enzymes (↑CAT in SWS, ↑SOD in NWS and SWS vs. adults) observed in this group may indicate a local reaction of the body to increased ROS production. It is well known that strengthening the antioxidant defense is a basic adaptive mechanism against ROS overproduction and oxidative stress [27, 30]. The oral cavity is a unique place—it is exposed to numerous environmental factors that induce the formation of free radicals. These include diet, stimulants, air pollutants, and dental materials [1, 2]. In our study, however, the resultant antioxidant capacity in older people was significantly reduced compared to adults (↓TAC and ↑TOS in saliva and plasma), which may suggest the exhaustion of systemic antioxidant reserves and greater sensitivity to oxidative damage associated with the process of aging. In our earlier studies, we have shown disturbances in a salivary antioxidant barrier and higher oxidative damage to proteins, lipids, and DNA in older people with dementia compared to age- and sex-matched controls [10, 11]. Thus, neuropsychiatric diseases can exacerbate the redox imbalance in older people.

A very interesting observation from our study is the fact that both local (↓SOD, ↓GSH, and ↓TAC in NWS and SWS) and systemic (↓GPx, ↓SOD, ↓UA, and ↓TAC in erythrocytes and plasma) antioxidant systems were less efficient in the group of children and adolescents (aged 2–14) than in the group of middle-aged people (45–65 years of age). The cause of these changes may be age-related immaturity of salivary/central mechanisms responsible for the synthesis and release of antioxidants. Indeed, Zalewska et al. demonstrated that the activity of Px and the synthesis of salivary nonspecific immunity proteins (such as lactoferrin and lysozyme) depend on the age of children and adolescents [31]. The inborn immune response is the first and quickest line of defense against microorganisms. Its purpose is to reduce the colonization of bacteria on teeth and mucous membranes and prevent the penetration of harmful substances into the body [31, 32]. Px and lactoferrin also act as free radical sweepers in the oral cavity. However, the increased activity of Px and CAT in NWS of children and adolescents observed in our study (compared to adults) may also suggest that some of the salivary antioxidant systems play a more important role at an early age than others. In fact, Px is considered the most significant antioxidant enzyme in saliva [32, 33]. The Px system not only participates in the decomposition of hydrogen peroxide (H_2_O_2_) but also catalyzes the oxidation of chloride ion Cl^−^ and the reduction of H_2_O_2_ to hypochlorous acid (HOCl), resulting in the formation of chloramines with a strong bactericidal effect. It should be noted that Px is the only antioxidant produced solely in salivary glands [1, 32]. Thus, a boost in Px activity indicates an increased rate of hydrogen peroxide removal in response to local changes in redox homeostasis. However, these hypotheses require further studies and clinical observations.

Disturbances in the antioxidant barrier predispose cell components to oxidative damage. In our study, the level of oxidative modification products of proteins (↑AGE, ↑AOPP) and lipids (↑MDA) was significantly higher in saliva and plasma of elderly people in comparison with middle-aged subjects and children. It is well known that the accumulation of oxidized protein aggregates plays a key role in the aging process [30, 34]. Actually, the functioning of lysosomes/proteasomes responsible for the distribution of damaged macromolecules weakens with age. This facilitates the accumulation of oxidized proteins, and the products of nonenzymatic glycosylation (AGE) are considered biological indicators of aging [10, 11, 29]. Interestingly, AGE combined with a specific receptor (RAGE) induce the formation of ROS by activating NADPH oxidase (NOX) that is the main source of free radicals in the cell. This, owing to positive feedback, intensifies redox imbalance and further production of free radicals, which is called ROS-induced ROS release [28, 30]. Also lipid peroxidation increases with the aging of the body. Lipid oxidation products interact with proteins or DNA, leading to cell death or uncontrolled proliferation [27, 29].

It is well known that about 60% of NWS is produced by submandibular glands, while the parotid glands produce about 25% of whole saliva [8, 35]. During stimulation, the share of the submandibular glands decreases (to approx. 30%) in favor of the parotid glands (approx. 60%). Thus, the composition of NWS and SWS is different (mainly in water, electrolyte, and protein content) [8, 35]; however, differences in the antioxidant profile of saliva are still unknown. Considering that the parotid glands are a highly aerobic organ [36, 37], it is not surprising that the oxidative processes in SWS are more intense than in NWS (↑TOS in SWS vs. NWS). Although we did not directly assess the rate of ROS production, the increased TOS level indicates a significantly higher content of free radicals and other oxidants in the stimulated salivary secretion. In our study, however, oxidative damage to proteins and lipids was generally more severe in NWS, which can be explained by the increased activity of the antioxidant enzymes in SWS (↑CAT in children and elderly people and ↑Px in adults and elderly people vs. NWS). Also, the TAC level was significantly higher in SWS compared to NWS, suggesting an adaptive response to increased ROS production in the parotid gland. It is believed that TAC characterizes the resultant antioxidant capacity of the biological system, taking into account the interactions between all enzymatic and nonenzymatic antioxidants [22]. Generally, no significant differences were found in the salivary redox profile of women and men in particular age groups; and consequently, the level of salivary oxidative stress parameters does not depend on gender but only on age.

Substances contained in saliva can be divided into two groups: compounds produced exclusively in salivary glands and those transported to saliva from plasma. It has been demonstrated that biomolecules can pass to the oral cavity through passive transport (diffusion and ultrafiltration), facilitated transport, pinocytosis, and active transport, so the concentration of some compounds in saliva can be even higher than in plasma/blood serum [7, 8]. Thus, saliva is a very attractive body fluid for diagnosing various diseases [12–14]. Other advantages of its use are noninvasive and painless collection, high durability of substances assayed in saliva, and economic reasons. Saliva is used to diagnose metabolic and infectious diseases, monitor the concentration of drugs, and detect drugs and stimulants [13, 38]. Moreover, the use of salivary oxidative stress biomarkers in the diagnosis of oral cavity diseases as well as systemic diseases is more and more often highlighted [13, 14, 39, 40]. The high diagnostic usefulness of salivary redox biomarkers has been demonstrated in patients with obesity [35, 41], cancer [42, 43], chronic kidney disease [15, 44], and dementia [10, 11]. However, little is still known about the relationship of salivary and blood redox homeostasis. Therefore, an important part of the presented study was the evaluation of correlations between oxidative stress parameters in NWS, SWS, plasma, and erythrocytes. Although antioxidants and protein/lipid oxidation products can be both synthesized in salivary glands and transported to the oral cavity from blood, in our study only the concentration of salivary oxidative modification products correlated with their content in plasma. This fact is not surprising as saliva is the first line of defense of the body against oxygen free radicals [1, 4]. Therefore, the salivary antioxidant barrier does not have to reflect central redox homeostasis. However, given the growing popularity of saliva in laboratory diagnostics, salivary products of protein and lipid oxidation may be potential biomarkers of systemic disorders. In fact, the diagnostic utility of AGE, AOPP, or MDA has been demonstrated in the diagnosis of nonalcoholic fatty liver disease [45], dementia [10, 11], chronic kidney disease [15], and obesity [35, 41]. Thus, saliva can be an alternative to blood or cerebrospinal fluid.

Factors regulating the volume and composition of the secreted saliva include age, gender, health status, and the type of stimulant [7, 46]. It has been shown that relatively low saliva secretion in newborns increases with age and reaches its first peak before the end of the 10th year of life [9]. After the age of 30, a downward trend in saliva secretion is observed [9], which is consistent with the results of our study. Interestingly, the concentration of salivary products of protein (AGE) and lipid (MDA) oxidation correlates negatively with the decrease of NWS and SWS secretion, which may suggest the contribution of oxidative stress to disturbing the secretory function of parotid and submandibular glands—as confirmed by the results of our previous studies [10, 11]. Salivary gland dysfunction is also evidenced by a decrease in total protein concentration in saliva [33, 47]. However, in the group of elderly people, total protein concentration was significantly higher compared to adults and children despite decreased secretion of NWS and SWS of the former. The observed increase in salivary protein content may be a consequence of a considerable drop in the volume of secreted saliva, which is referred to as “saliva concentration” [11, 48].

Finally, it needs to be mentioned that our work also had certain limitations. Despite restrictive inclusion/exclusion criteria, the study involved elderly people with hypertension and ischemic heart disease, i.e., diseases common in this age group. Thus, when describing the redox homeostasis of older people, we should consider not only aging mechanisms but also the inseparable disease process. Moreover, we have evaluated some selected biomarkers of oxidative stress, which makes it impossible to fully compare the redox homeostasis of children, adults, and the elderly. We are also aware of the imperfect division of the study groups, which requires further research in wider age ranges. However, our study was the first to characterize the redox balance of saliva and blood in different age groups, although it should be pointed out that further research is needed in this area.

## 5. Conclusions

1. The antioxidant barrier of saliva and blood is most effective in adults and generally decreases with age
2. The low efficiency of the antioxidant systems in children may indicate an age-related immaturity of defense mechanisms against oxidative stress and the overproduction of ROS
3. The concentration of the products of protein and lipid oxidative damage in nonstimulated saliva generally reflects their content in plasma
4. The level of oxidative stress biomarkers in children, adults, and the elderly generally does not vary based on gender

## Figures and Tables

**Table 1:** Clinical characteristics and dental examination of healthy children (aged 2-14), adults (aged 25-45), and elderly people (aged 65-85).

	2-14		25-45		65-85		ANOVA *p*
	Men (*n* = 15)	Female (*n* = 15)	Men (*n* = 15)	Female (*n* = 15)	Men (*n* = 15)	Female (*n* = 15)	
Age	7.4 ± 2.8	8.1 ± 2.4	37.4 ± 5.8	39.5 ± 4.2	78.2 ± 6.5	79.6 ± 4.4	NA
*Blood count and biochemical tests*							
RBC (10^6^/*μ*L)	4.3 ± 0.3	4 ± 0.1	4.7 ± 0.2	4.4 ± 0.2	4.5 ± 0.4	4.1 ± 0.1	NS
Hb (g/dL)	14.3 ± 0.2	13.9 ± 0.2	15.2 ± 0.9	13.2 ± 0.9	14.1 ± 0.5	13.9 ± 0.1	NS
HCT (%)	39.5 ± 0.7	39.3 ± 0.5	43.5 ± 4.1	40.2 ± 4.5	39 ± 1.2	38.2 ± 0.9	NS
WBC (10^3^/*μ*L)	7.5 ± 0.6	7.7 ± 0.4	5.9 ± 1.7	5.3 ± 2.1	7.4 ± 0.4	7.3 ± 0.7	NS
PLT (10^3^/*μ*L)	367 ± 17	358.2 ± 11	328.3 ± 16	344.3 ± 19	249 ± 14	256.6 ± 18	NS
CRP (mg/L)	1.2 ± 0.8	1.7 ± 1	1.5 ± 1.1	1.8 ± 0.7	2.9 ± 1.1	2.8 ± 0.9	NS
Na^+^ (mmol/L)	ND	ND	142.2 ± 1.5	140.2 ± 0.9	139.2 ± 0.8	140 ± 1.1	NS
K^+^ mmol/L)	ND	ND	4.4 ± 0.1	4.5 ± 0.1	4.2 ± 0.1	4.2 ± 0.1	NS
ALT (U/L)	ND	ND	22.3 ± 8.2	19.8 ± 6.1	28.2 ± 4.2	27.7 ± 6.3	NS
Creatinine (mg/dL)	0.8 ± 0.2	0.7 ± 0.1	0.8 ± 0.1	0.8 ± 0.1	0.9 ± 0.1	0.9 ± 0.1	NS
*Systemic diseases and medications*							
Hypertension, *n* (%)	0 (0)	0 (0)	1 (6.7)	2 (13.3)	3 (20)	3 (20)	NA
Type 2 diabetes, *n* (%)	0 (0)	0 (0)	1 (6.7)	1 (6.7)	2 (13.3)	2 (13.3)	NA
Coronary artery disease, *n* (%)	0 (0)	0 (0)	1 (6.7)	1 (6.7)	1 (6.7)	2 (13.3)	NA
Atherosclerosis, *n* (%)	0 (0)	0 (0)	0 (0)	0 (0)	2 (13.3)	2 (13.3)	NA
Osteoporosis, *n* (%)	0 (0)	0 (0)	0 (0)	1 (6.7)	2 (13.3)	2 (13.3)	NA
<5 drugs/day, *n* (%)	0 (0)	0 (0)	3 (20)	3 (20)	4 (26.7)	5 (33.3)	NA
≥5 drugs/day, *n* (%)	0 (0)	0 (0)	0 (0)	0 (0)	2 (13.3)	2 (13.3)	NA
*Dental examination*							
NWS flow (mL/min)	0.41 ± 0.04	0.41 ± 0.05	0.49 ± 0.05	0.47 ± 0.06	0.25 ± 0.09	0.28 ± 0.1	**<0.001**
SWS flow (mL/min)	1.4 ± 0.1	1.4 ± 0.1	2 ± 0.1	1.9 ± 0.1	1.2 ± 0.1	1.3 ± 0.1	**<0.001**
TSP NWS (*μ*g/mL)	1342 ± 299	1344 ± 155	1192 ± 506	1276 ± 624	2298 ± 665	2406 ± 709	**<0.001**
TSP SWS (*μ*g/mL)	1023 ± 322	1006 ± 303	1130 ± 272	1231 ± 308	1999 ± 904	2125 ± 860	**<0.001**
DMFT	3.1 ± 0.8	3 ± 0.2	17.7 ± 4.2	15.2 ± 3.8	30.1 ± 0.1	30.5 ± 0.1	**<0.001**
Dmft	11.1 ± 0.8	10.5 ± 0.9	NA	NA	NA	NA	NA
PBI	0.0 ± 0.2	0.0 ± 0.3	0.5 ± 0.2	0.4 ± 0.2	1.5 ± 0.3	1.5 ± 0.2	**<0.001**
GI	0.0 ± 0.4	0.0 ± 0.3	0.3 ± 0.2	0.3 ± 0.1	1.7 ± 0.5	1.4 ± 0.2	**<0.001**

ALT—alanine transferase; CRP—C-reactive protein; dmft—decayed, missing, filled teeth for primary teeth; DMFT—decayed, missing, filled teeth for permanent teeth; GI—Gingival Index; Hb—hemoglobin; HCT—hematocrit; K^+^—potassium; Na^+^—sodium; NA—not applicable; ND—no data; NS—no significance; NWS—nonstimulated whole saliva; PBI—Papilla Bleeding Index; PLT—platelets; RBC—red blood cells; SWS—stimulated whole saliva; TSP—total salivary protein; WBC—white blood cells.

**Table 2:** Differences between nonstimulated and stimulated saliva of healthy children (aged 2-14), adults (aged 25-45), and elderly people (aged 65-85).

	2-14		25-45		65-85	
	NWS	SWS	NWS	SWS	NWS	SWS
CAT (mU/mg protein)	0.93 ± 0.09	0.52 ± 0.05^∗^	0.41 ± 0.04	0.36 ± 0.05	0.47 ± 0.05	0.59 ± 0.03^∗^
Px (nmol/min/mg protein)	0.45 ± 0.02	0.41 ± 0.02	0.29 ± 0.02	0.41 ± 0.04^∗^	0.18 ± 0.02	0.28 ± 0.011^∗^
SOD (mU/mg protein)	0.5 ± 0.04	0.55 ± 0.04	1.4 ± 0.1	1.8 ± 0.1^∗^	3.5 ± 0.2	2.3 ± 0.07^∗^
GSH (*μ*g/mg protein)	0.64 ± 0.1	1.6 ± 0.1^∗^	3.9 ± 0.3	2.6 ± 0.1^∗^	2.6 ± 0.09	2.2 ± 0.1^∗^
UA (*μ*g/mg protein)	3.3 ± 0.2	2.9 ± 0.2	5 ± 0.2	3.5 ± 0.3^∗^	1.9 ± 0.2	2.4 ± 0.2
TAC (*μ*mol/mg protein)	0.48 ± 0.03	0.71 ± 0.04^∗^	1.2 ± 0.06	1.3 ± 0.05	0.3 ± 0.01	0.57 ± 0.04^∗^
TOS (nmol/mg protein)	6.6 ± 0.5	20 ± 1.3^∗^	9.8 ± 0.9	30 ± 1.8^∗^	13 ± 0.9	41 ± 2.3^∗^
AGE (AFU/mg protein)	1.4 ± 0.09	0.82 ± 0.08^∗^	1.1 ± 0.1	0.98 ± 0.05	2.1 ± 0.1	1.2 ± 0.05^∗^
AOPP (nmol/mg protein)	23 ± 1.6	14 ± 1.1^∗^	32 ± 3.2	14 ± 1.3^∗^	44 ± 2.9	29 ± 2.9
MDA (*μ*mol/mg protein)	87 ± 4.7	60 ± 4.4^∗^	247 ± 16	410 ± 35^∗^	693 ± 59	623 ± 39

AGE—advanced glycation end products; AOPP—advanced oxidation protein products; CAT—catalase; GPx—glutathione peroxidase; GSH—reduced glutathione; MDA—malondialdehyde; NWS—nonstimulated whole saliva; Px—salivary peroxidase; SOD—superoxide dismutase-1; SWS—stimulated whole saliva; TAC—total antioxidant capacity; TOS—advanced oxidation protein products; UA—uric acid. ^∗^*p* < 0.05 vs. NWS.

## Data Availability

The article contains complete data used to support the findings of this study.

## Supplementary Materials

Supplementary Materials Table 1: *r* value of correlations between nonstimulated saliva (NWS) and plasma/erythrocyte redox biomarkers in healthy children aged 2-14. Table 2: *p* value of correlations between nonstimulated saliva (NWS) and plasma/erythrocyte redox biomarkers in healthy children aged 2-14. Table 3: *r* value of correlations between stimulated saliva (SWS) and plasma/erythrocyte redox biomarkers in healthy children aged 2-14. Table 4: *p* value of correlations between stimulated saliva (SWS) and plasma/erythrocyte redox biomarkers in healthy children aged 2-14. Table 5: *r* value of correlations between nonstimulated saliva (NWS) and plasma/erythrocyte redox biomarkers in healthy adults aged 25-45. Table 6: *p* value of correlations between nonstimulated saliva (NWS) and plasma/erythrocyte redox biomarkers in healthy adults aged 25-45. Table 7: *r* value of correlations between stimulated saliva (SWS) and plasma/erythrocyte redox biomarkers in healthy adults aged 25-45. Table 8: *p* value of correlations between stimulated saliva (SWS) and plasma/erythrocyte redox biomarkers in healthy adults aged 25-45. Table 9: *r* value of correlations between nonstimulated saliva (NWS) and plasma/erythrocyte redox biomarkers in healthy elderly people aged 65-85. Table 10: *p* value of correlations between nonstimulated saliva (NWS) and plasma/erythrocyte redox biomarkers in healthy elderly people aged 65-85. Table 11: *r* value of correlations between stimulated saliva (SWS) and plasma/erythrocyte redox biomarkers in healthy elderly people aged 65-85. Table 12: *p* value of correlations between stimulated saliva (SWS) and plasma/erythrocyte redox biomarkers in healthy elderly people aged 65-85.
