# Supplementary material for: Salivary Antioxidant Barrier, Redox Status, and Oxidative Damage to Proteins and Lipids in Healthy Children, Adults, and the Elderly
Source: Oxid Med Cell Longev. 2019 Dec 5;2019:4393460. doi: 10.1155/2019/4393460 (PMC6915128; doi:10.1155/2019/4393460)
Supplement: Supplementary Materials — Table 1: r value of correlations between nonstimulated saliva (NWS) and plasma/erythrocyte redox biomarkers in healthy children aged 2-14. Table 2: p value of correlations between nonstimulated saliva (NWS) and plasma/erythrocyte redox biomarkers in healthy children aged 2-14. Table 3: r value of correlations between stimulated saliva (SWS) and plasma/erythrocyte redox biomarkers in healthy children aged 2-14. Table 4: p value of correlations between stimulated saliva (SWS) and plasma/erythrocyte redox biomarkers in healthy children aged 2-14. Table 5: r value of correlations between nonstimulated saliva (NWS) and plasma/erythrocyte redox biomarkers in healthy adults aged 25-45. Table 6: p value of correlations between nonstimulated saliva (NWS) and plasma/erythrocyte redox biomarkers in healthy adults aged 25-45. Table 7: r value of correlations between stimulated saliva (SWS) and plasma/erythrocyte redox biomarkers in healthy adults aged 25-45. Table 8: p value of correlations between stimulated saliva (SWS) and plasma/erythrocyte redox biomarkers in healthy adults aged 25-45. Table 9: r value of correlations between nonstimulated saliva (NWS) and plasma/erythrocyte redox biomarkers in healthy elderly people aged 65-85. Table 10: p value of correlations between nonstimulated saliva (NWS) and plasma/erythrocyte redox biomarkers in healthy elderly people aged 65-85. Table 11: r value of correlations between stimulated saliva (SWS) and plasma/erythrocyte redox biomarkers in healthy elderly people aged 65-85. Table 12: p value of correlations between stimulated saliva (SWS) and plasma/erythrocyte redox biomarkers in healthy elderly people aged 65-85. [file 4393460.f1.pdf]

**Table 1. R value of correlations between non-stimulated saliva (NWS) and plasma/erythrocytes redox biomarkers in healthy children aged 2-14.**

| | CAT erythrocytes | GPx erythrocytes | SOD erythrocytes | GSH plasma | UA plasma | TAC plasma | TOS plasma | AGE plasma | AOPP plasma | MDA plasma |
|---|---|---|---|---|---|---|---|---|---|---|
| CAT NWS | -0.003 | -0.049 | 0.045 | -0.03 | 0.037 | -0.287 | 0.006 | 0.27 | -0.261 | 0.249 |
| Px NWS | -0.137 | -0.148 | 0.065 | -0.081 | -0.065 | -0.264 | 0.158 | 0.041 | 0.109 | -0.028 |
| SOD NWS | 0.247 | -0.1 | 0.012 | 0.155 | -0.19 | 0.229 | -0.069 | 0.136 | -0.041 | 0.283 |
| GSH NWS | 0.018 | 0.173 | 0.711 | 0.217 | -0.047 | -0.264 | 0.072 | -0.334 | -0.274 | -0.287 |
| UA NWS | 0.141 | -0.048 | -0.403 | -0.217 | -0.221 | -0.104 | -0.121 | 0.126 | 0.036 | 0.226 |
| TAC NWS | -0.032 | 0.026 | 0.157 | 0.327 | -0.096 | -0.172 | -0.283 | 0.03 | -0.075 | -0.361 |
| TOS NWS | 0.009 | 0.266 | 0.281 | 0.034 | -0.212 | 0.036 | 0.124 | -0.097 | -0.193 | 0.021 |
| AGE NWS | -0.03 | -0.062 | -0.001 | 0.029 | -0.387 | 0.029 | 0.052 | 0.637 | 0.03 | 0.147 |
| AOPP NWS | -0.304 | -0.038 | -0.133 | 0.225 | -0.106 | -0.126 | -0.079 | 0.27 | 0.548 | 0.341 |
| MDA NWS | -0.102 | -0.085 | -0.216 | -0.082 | -0.35 | -0.054 | -0.093 | 0.307 | -0.005 | 0.541 |

**Table 2. P value of correlations between non-stimulated saliva (NWS) and plasma/erythrocytes redox biomarkers in healthy children aged 2-14.**

| | CAT erythrocytes | GPx erythrocytes | SOD erythrocytes | GSH plasma | UA plasma | TAC plasma | TOS plasma | AGE plasma | AOPP plasma | MDA plasma |
|---|---|---|---|---|---|---|---|---|---|---|
| CAT NWS | 0.986 | 0.798 | 0.813 | 0.874 | 0.845 | 0.125 | 0.976 | 0.15 | 0.164 | 0.185 |
| Px NWS | 0.469 | 0.436 | 0.733 | 0.669 | 0.733 | 0.158 | 0.403 | 0.828 | 0.565 | 0.885 |
| SOD NWS | 0.189 | 0.599 | 0.951 | 0.414 | 0.315 | 0.224 | 0.715 | 0.473 | 0.831 | 0.13 |
| GSH NWS | 0.924 | 0.361 | 1.06E-05 | 0.249 | 0.805 | 0.159 | 0.707 | 0.071 | 0.142 | 0.124 |
| UA NWS | 0.458 | 0.8 | 0.027 | 0.25 | 0.24 | 0.584 | 0.523 | 0.505 | 0.85 | 0.231 |
| TAC NWS | 0.866 | 0.891 | 0.408 | 0.077 | 0.613 | 0.364 | 0.129 | 0.873 | 0.695 | 0.05 |
| TOS NWS | 0.962 | 0.155 | 0.133 | 0.858 | 0.26 | 0.851 | 0.515 | 0.611 | 0.307 | 0.91 |
| AGE NWS | 0.875 | 0.744 | 0.998 | 0.878 | 0.034 | 0.881 | 0.785 | 1.53E-04 | 0.874 | 0.438 |
| AOPP NWS | 0.103 | 0.841 | 0.485 | 0.233 | 0.576 | 0.508 | 0.678 | 0.15 | 0.002 | 0.065 |
| MDA NWS | 0.591 | 0.654 | 0.252 | 0.666 | 0.058 | 0.776 | 0.625 | 0.099 | 0.978 | 0.002 |

**Table 3. R value of correlations between stimulated saliva (SWS) and plasma/erythrocytes redox biomarkers in healthy children aged 2-14.**

| | CAT erythrocytes | GPx erythrocytes | SOD erythrocytes | GSH plasma | UA plasma | TAC plasma | TOS plasma | AGE plasma | AOPP plasma | MDA plasma |
|---|---|---|---|---|---|---|---|---|---|---|
| CAT SWS | -0.272 | 0.254 | 0.167 | -0.213 | -0.223 | 0.044 | -0.194 | 0.202 | 0.018 | 0.18 |
| Px SWS | 0.129 | 0.007 | 0.062 | -0.446 | -0.053 | 0.195 | 0.029 | -0.373 | -0.011 | -0.036 |
| SOD SWS | 0.181 | -0.142 | -0.086 | -0.348 | -0.013 | 0.263 | -0.067 | -0.161 | 0.144 | 0.207 |
| GSH SWS | -0.102 | 0.023 | 0.161 | -0.085 | -0.433 | 0.298 | -0.102 | 0.022 | -0.359 | 0.067 |
| UA SWS | 0.079 | -0.369 | -0.04 | -0.072 | 0.187 | -0.113 | -0.036 | -0.216 | -0.254 | 0.055 |
| TAC SWS | -0.476 | -0.016 | 0.262 | 0.248 | 0.053 | -0.454 | -0.098 | -0.16 | -0.035 | -0.001 |
| TOS SWS | 0.233 | -0.508 | -0.291 | -0.208 | 0.131 | 0.085 | -0.047 | -0.129 | 0.071 | -0.072 |
| AGE SWS | 0.157 | -0.354 | 0.148 | -0.479 | -0.122 | 0.001 | -0.084 | -0.198 | -0.271 | -0.108 |
| AOPP SWS | 0.096 | -0.092 | -0.087 | 0.003 | -0.083 | 0.193 | -0.089 | 0.02 | -0.041 | 0.357 |
| MDA SWS | 0.177 | 0.216 | -0.028 | -0.276 | -0.13 | 0.237 | -0.131 | -0.136 | -0.077 | 0.194 |

**Table 4. P value of correlations between stimulated saliva (SWS) and plasma/erythrocytes redox biomarkers in healthy children aged 2-14.**

| | CAT erythrocytes | GPx erythrocytes | SOD erythrocytes | GSH plasma | UA plasma | TAC plasma | TOS plasma | AGE plasma | AOPP plasma | MDA plasma |
|---|---|---|---|---|---|---|---|---|---|---|
| CAT SWS | 0.146 | 0.175 | 0.379 | 0.259 | 0.237 | 0.816 | 0.304 | 0.284 | 0.923 | 0.341 |
| Px SWS | 0.499 | 0.97 | 0.747 | 0.014 | 0.782 | 0.301 | 0.879 | 0.042 | 0.956 | 0.852 |
| SOD SWS | 0.34 | 0.455 | 0.651 | 0.06 | 0.944 | 0.161 | 0.725 | 0.396 | 0.446 | 0.273 |
| GSH SWS | 0.593 | 0.906 | 0.395 | 0.655 | 0.017 | 0.11 | 0.591 | 0.908 | 0.051 | 0.725 |
| UA SWS | 0.678 | 0.045 | 0.834 | 0.704 | 0.323 | 0.554 | 0.85 | 0.251 | 0.176 | 0.772 |
| TAC SWS | 0.008 | 0.933 | 0.162 | 0.187 | 0.78 | 0.012 | 0.608 | 0.399 | 0.856 | 0.997 |
| TOS SWS | 0.216 | 0.004 | 0.119 | 0.271 | 0.489 | 0.655 | 0.806 | 0.495 | 0.71 | 0.705 |
| AGE SWS | 0.409 | 0.055 | 0.435 | 0.007 | 0.521 | 0.996 | 0.661 | 0.293 | 0.148 | 0.571 |
| AOPP SWS | 0.612 | 0.628 | 0.648 | 0.988 | 0.663 | 0.306 | 0.641 | 0.917 | 0.83 | 0.053 |
| MDA SWS | 0.35 | 0.252 | 0.882 | 0.141 | 0.493 | 0.206 | 0.491 | 0.475 | 0.686 | 0.305 |

**Table 5. R value of correlations between non-stimulated saliva (NWS) and plasma/erythrocytes redox biomarkers in healthy adults aged 25-45.**

| | CAT erythrocytes | GPx erythrocytes | SOD erythrocytes | GSH plasma | UA plasma | TAC plasma | TOS plasma | AGE plasma | AOPP plasma | MDA plasma |
|---|---|---|---|---|---|---|---|---|---|---|
| CAT NWS | 0.074 | 0.094 | -0.287 | -0.09 | -0.277 | 0.052 | -0.102 | 0.273 | 0.059 | 0.071 |
| Px NWS | -0.018 | 0.232 | 0.259 | -0.12 | -0.232 | 0.358 | -0.224 | 0.178 | -0.054 | -0.003 |
| SOD NWS | 0.038 | 0.005 | -0.052 | -0.102 | -0.178 | -0.365 | -0.162 | 0.249 | -0.136 | 0.05 |
| GSH NWS | 0.269 | 0.02 | -0.041 | -0.016 | 0.047 | -0.014 | 0.01 | -0.018 | -0.292 | 0.259 |
| UA NWS | 0.223 | -0.139 | 0.334 | -0.269 | 0.35 | -0.203 | 0.437 | -0.267 | -0.222 | -0.109 |
| TAC NWS | 0.019 | -0.055 | 0.185 | 0.41 | 0.03 | -0.191 | -0.198 | -0.159 | 0.07 | 0.303 |
| TOS NWS | 0.121 | 0.053 | -0.105 | 0.097 | -0.288 | 0.191 | -0.153 | 0.116 | -0.082 | 0.205 |
| AGE NWS | 0.13 | 0.219 | 0.104 | -0.142 | -0.234 | 0.106 | 0.022 | 0.586 | 0.082 | 0.015 |
| AOPP NWS | 0.042 | -0.168 | 0.067 | 0.135 | -0.014 | -0.074 | 0.085 | 0.006 | 0.547 | -0.042 |
| MDA NWS | -0.01 | -0.191 | 0.118 | -0.338 | -0.015 | 0.118 | 0.155 | -0.033 | -0.407 | 0.318 |

**Table 6. P value of correlations between non-stimulated saliva (NWS) and plasma/erythrocytes redox biomarkers in healthy adults aged 25-45.**

| | CAT erythrocytes | GPx erythrocytes | SOD erythrocytes | GSH plasma | UA plasma | TAC plasma | TOS plasma | AGE plasma | AOPP plasma | MDA plasma |
|---|---|---|---|---|---|---|---|---|---|---|
| CAT NWS | 0.696 | 0.623 | 0.124 | 0.637 | 0.139 | 0.784 | 0.593 | 0.144 | 0.756 | 0.709 |
| Px NWS | 0.925 | 0.218 | 0.167 | 0.526 | 0.217 | 0.052 | 0.234 | 0.348 | 0.775 | 0.988 |
| SOD NWS | 0.844 | 0.978 | 0.785 | 0.592 | 0.346 | 0.047 | 0.394 | 0.184 | 0.474 | 0.792 |
| GSH NWS | 0.151 | 0.917 | 0.829 | 0.931 | 0.806 | 0.941 | 0.96 | 0.924 | 0.118 | 0.167 |
| UA NWS | 0.236 | 0.464 | 0.071 | 0.151 | 0.058 | 0.283 | 0.016 | 0.153 | 0.239 | 0.566 |
| TAC NWS | 0.922 | 0.774 | 0.327 | 0.024 | 0.875 | 0.312 | 0.295 | 0.401 | 0.715 | 0.104 |
| TOS NWS | 0.523 | 0.783 | 0.582 | 0.61 | 0.122 | 0.311 | 0.42 | 0.54 | 0.666 | 0.278 |
| AGE NWS | 0.492 | 0.245 | 0.584 | 0.455 | 0.213 | 0.579 | 0.908 | 0.001 | 0.668 | 0.938 |
| AOPP NWS | 0.825 | 0.374 | 0.727 | 0.478 | 0.94 | 0.696 | 0.654 | 0.973 | 0.002 | 0.826 |
| MDA NWS | 0.959 | 0.313 | 0.533 | 0.067 | 0.935 | 0.536 | 0.413 | 0.863 | 0.026 | 0.087 |

**Table 7. R value of correlations between stimulated saliva (SWS) and plasma/erythrocytes redox biomarkers in healthy adults aged 25-45.**

| | CAT erythrocytes | GPx erythrocytes | SOD erythrocytes | GSH plasma | UA plasma | TAC plasma | TOS plasma | AGE plasma | AOPP plasma | MDA plasma |
|---|---|---|---|---|---|---|---|---|---|---|
| CAT SWS | -0.035 | 0.222 | -0.243 | -0.211 | -0.088 | 0.131 | -0.049 | 0.441 | -0.208 | 0.387 |
| Px SWS | -0.113 | -0.191 | 0.155 | -0.272 | 0.216 | 0.084 | 0.273 | 0.049 | -0.047 | -0.046 |
| SOD SWS | 0.303 | -0.037 | -0.161 | -0.153 | -0.208 | -0.138 | -0.107 | 0.321 | -0.052 | 0.1 |
| GSH SWS | 0.036 | 0.12 | 0.063 | -0.172 | -0.203 | -0.153 | -0.171 | 0.011 | -0.125 | -0.002 |
| UA SWS | -0.255 | 0.086 | -0.199 | -0.099 | 0.087 | 0.241 | 0.045 | -0.017 | 0.002 | 0.033 |
| TAC SWS | 0.055 | -0.001 | -0.049 | 0.039 | 0.236 | 0.193 | 0.306 | -0.173 | -0.103 | -0.117 |
| TOS SWS | 0.175 | -0.068 | -0.17 | 0.164 | -0.229 | 0.091 | -0.085 | 0.07 | -0.011 | 0.255 |
| AGE SWS | 0.111 | 0.047 | 0.229 | 0.021 | -0.076 | -0.035 | 0.016 | 0.248 | 0.195 | -0.188 |
| AOPP SWS | -0.107 | -0.148 | 0.133 | 0.038 | -0.259 | 0.074 | -0.059 | 0.102 | 0.18 | -0.35 |
| MDA SWS | 0.107 | -0.086 | -0.327 | -0.002 | 0.077 | -0.045 | 0.243 | 0.189 | -0.124 | 0.55 |

**Table 8. P value of correlations between stimulated saliva (SWS) and plasma/erythrocytes redox biomarkers in healthy adults aged 25-45.**

| | CAT erythrocytes | GPx erythrocytes | SOD erythrocytes | GSH plasma | UA plasma | TAC plasma | TOS plasma | AGE plasma | AOPP plasma | MDA plasma |
|---|---|---|---|---|---|---|---|---|---|---|
| CAT SWS | 0.855 | 0.238 | 0.196 | 0.263 | 0.645 | 0.489 | 0.795 | 0.015 | 0.27 | 0.035 |
| Px SWS | 0.558 | 0.321 | 0.423 | 0.153 | 0.261 | 0.665 | 0.151 | 0.8 | 0.81 | 0.812 |
| SOD SWS | 0.104 | 0.845 | 0.395 | 0.419 | 0.269 | 0.467 | 0.572 | 0.083 | 0.786 | 0.6 |
| GSH SWS | 0.85 | 0.528 | 0.739 | 0.363 | 0.283 | 0.419 | 0.365 | 0.953 | 0.51 | 0.992 |
| UA SWS | 0.174 | 0.652 | 0.291 | 0.603 | 0.646 | 0.199 | 0.813 | 0.928 | 0.991 | 0.861 |
| TAC SWS | 0.772 | 0.997 | 0.795 | 0.84 | 0.209 | 0.308 | 0.1 | 0.362 | 0.588 | 0.538 |
| TOS SWS | 0.355 | 0.722 | 0.37 | 0.386 | 0.224 | 0.631 | 0.654 | 0.713 | 0.954 | 0.174 |
| AGE SWS | 0.559 | 0.807 | 0.223 | 0.912 | 0.689 | 0.853 | 0.935 | 0.187 | 0.301 | 0.321 |
| AOPP SWS | 0.575 | 0.435 | 0.483 | 0.842 | 0.166 | 0.696 | 0.757 | 0.591 | 0.342 | 0.058 |
| MDA SWS | 0.574 | 0.653 | 0.078 | 0.991 | 0.685 | 0.814 | 0.196 | 0.317 | 0.514 | 0.002 |

**Table 9. R value of correlations between non-stimulated saliva (NWS) and plasma/erythrocytes redox biomarkers in healthy elderly people aged 65-85.**

| | CAT erythrocytes | GPx erythrocytes | SOD erythrocytes | GSH plasma | UA plasma | TAC plasma | TOS plasma | AGE plasma | AOPP plasma | MDA plasma |
|---|---|---|---|---|---|---|---|---|---|---|
| CAT NWS | -0.347 | 0.307 | 0.25 | -0.059 | 0.269 | 0.059 | 0.05 | 0.004 | 0.191 | 0.015 |
| Px NWS | -0.176 | -0.096 | -0.01 | -0.15 | 0.015 | 0.454 | 0.273 | -0.12 | -0.252 | 0.159 |
| SOD NWS | -0.009 | -0.095 | 0.179 | 0.187 | 0.189 | 0.062 | -0.344 | 0.034 | -0.081 | -0.053 |
| GSH NWS | -0.303 | 0.303 | 0.27 | -0.016 | -0.047 | -0.284 | 0.192 | 0.03 | 0.035 | 0.004 |
| UA NWS | -0.037 | -0.438 | 0.123 | 0.48 | -0.108 | -0.071 | -0.355 | -0.19 | 0.281 | 0.052 |
| TAC NWS | 0.173 | 0.113 | 0.255 | -0.144 | 0.031 | -0.194 | 0.385 | 0.256 | -0.228 | -0.138 |
| TOS NWS | -0.011 | -0.065 | 0.142 | -0.002 | 0.07 | 0.243 | -0.45 | -0.101 | -0.066 | 0.435 |
| AGE NWS | 0.189 | -0.069 | 0.148 | 0.152 | 0.155 | 0.044 | -0.281 | 0.394 | -0.072 | 0.176 |
| AOPP NWS | 0.067 | 0.043 | -0.269 | -0.006 | -0.223 | 0.121 | -0.157 | -0.136 | 0.698 | -0.139 |
| MDA NWS | 0.212 | -0.08 | 0.363 | -0.013 | -0.131 | 0.331 | -0.336 | -0.152 | 0.029 | 0.864 |

**Table 10. P value of correlations between non-stimulated saliva (NWS) and plasma/erythrocytes redox biomarkers in healthy elderly people aged 65-85.**

| | CAT erythrocytes | GPx erythrocytes | SOD erythrocytes | GSH plasma | UA plasma | TAC plasma | TOS plasma | AGE plasma | AOPP plasma | MDA plasma |
|---|---|---|---|---|---|---|---|---|---|---|
| CAT NWS | 0.061 | 0.099 | 0.182 | 0.758 | 0.15 | 0.755 | 0.793 | 0.985 | 0.312 | 0.935 |
| Px NWS | 0.351 | 0.614 | 0.957 | 0.43 | 0.937 | 0.012 | 0.145 | 0.529 | 0.18 | 0.401 |
| SOD NWS | 0.962 | 0.619 | 0.343 | 0.322 | 0.317 | 0.746 | 0.063 | 0.86 | 0.671 | 0.782 |
| GSH NWS | 0.103 | 0.104 | 0.149 | 0.934 | 0.805 | 0.129 | 0.311 | 0.876 | 0.856 | 0.982 |
| UA NWS | 0.847 | 0.016 | 0.518 | 0.007 | 0.57 | 0.709 | 0.054 | 0.314 | 0.132 | 0.786 |
| TAC NWS | 0.36 | 0.551 | 0.174 | 0.449 | 0.87 | 0.304 | 0.036 | 0.172 | 0.227 | 0.466 |
| TOS NWS | 0.955 | 0.733 | 0.456 | 0.994 | 0.714 | 0.196 | 0.013 | 0.594 | 0.73 | 0.016 |
| AGE NWS | 0.316 | 0.715 | 0.436 | 0.423 | 0.414 | 0.819 | 0.133 | 0.031 | 0.705 | 0.353 |
| AOPP NWS | 0.727 | 0.821 | 0.15 | 0.974 | 0.236 | 0.523 | 0.409 | 0.472 | 1.79E-05 | 0.464 |
| MDA NWS | 0.26 | 0.674 | 0.048 | 0.947 | 0.489 | 0.074 | 0.07 | 0.423 | 0.879 | 7.52E-10 |

**Table 11. R value of correlations between stimulated saliva (SWS) and plasma/erythrocytes redox biomarkers in healthy elderly people aged 65-85.**

| | CAT erythrocytes | GPx erythrocytes | SOD erythrocytes | GSH plasma | UA plasma | TAC plasma | TOS plasma | AGE plasma | AOPP plasma | MDA plasma |
|---|---|---|---|---|---|---|---|---|---|---|
| CAT SWS | -0.331 | 0.174 | 0.023 | 0.34 | 0.125 | 0.113 | -0.167 | 0.105 | -0.061 | -0.16 |
| Px SWS | 0.146 | 0.208 | -0.231 | -0.203 | -0.141 | -0.084 | 0.06 | -0.409 | 0.303 | -0.196 |
| SOD SWS | -0.085 | -0.097 | 0.055 | -0.085 | 0.163 | 0.274 | -0.092 | -0.09 | -0.055 | -0.194 |
| GSH SWS | -0.068 | -0.089 | 0.255 | 0.161 | 0.139 | -0.264 | 0.322 | -0.025 | -0.1 | 0.073 |
| UA SWS | 0.261 | -0.161 | 0.049 | 0.421 | -0.008 | 0.079 | -0.145 | -0.094 | 0.113 | 0.162 |
| TAC SWS | 0.369 | -0.16 | 0.051 | -0.354 | 0.115 | 0.295 | 0.146 | -0.334 | -0.118 | -0.348 |
| TOS SWS | -0.181 | 0.179 | -0.125 | -0.047 | -0.288 | -0.05 | -0.112 | -0.021 | -0.244 | -0.064 |
| AGE SWS | -0.4 | 0.392 | -0.019 | -0.192 | 0.042 | -0.193 | 0.276 | 0.098 | -0.304 | -0.033 |
| AOPP SWS | 0.06 | -0.204 | 0.291 | 0.289 | 0.138 | -0.09 | -0.163 | 0.154 | -0.142 | -0.017 |
| MDA SWS | -0.2 | -0.149 | -0.196 | 0.118 | 0.257 | 0.097 | 0.151 | -0.072 | -0.23 | 0.507 |

**Table 12. P value of correlations between stimulated saliva (SWS) and plasma/erythrocytes redox biomarkers in healthy elderly people aged 65-85.**

| | CAT erythrocytes | GPx erythrocytes | SOD erythrocytes | GSH plasma | UA plasma | TAC plasma | TOS plasma | AGE plasma | AOPP plasma | MDA plasma |
|---|---|---|---|---|---|---|---|---|---|---|
| CAT SWS | 0.074 | 0.359 | 0.902 | 0.066 | 0.512 | 0.552 | 0.377 | 0.581 | 0.747 | 0.399 |
| Px SWS | 0.44 | 0.27 | 0.22 | 0.282 | 0.458 | 0.659 | 0.751 | 0.025 | 0.104 | 0.3 |
| SOD SWS | 0.655 | 0.61 | 0.771 | 0.655 | 0.39 | 0.143 | 0.629 | 0.637 | 0.771 | 0.304 |
| GSH SWS | 0.721 | 0.64 | 0.174 | 0.396 | 0.463 | 0.159 | 0.083 | 0.895 | 0.599 | 0.702 |
| UA SWS | 0.163 | 0.395 | 0.797 | 0.021 | 0.968 | 0.677 | 0.446 | 0.621 | 0.551 | 0.391 |
| TAC SWS | 0.045 | 0.398 | 0.79 | 0.055 | 0.545 | 0.114 | 0.441 | 0.072 | 0.533 | 0.06 |
| TOS SWS | 0.34 | 0.345 | 0.511 | 0.805 | 0.123 | 0.791 | 0.556 | 0.912 | 0.194 | 0.737 |
| AGE SWS | 0.028 | 0.032 | 0.921 | 0.31 | 0.826 | 0.306 | 0.14 | 0.606 | 0.103 | 0.863 |
| AOPP SWS | 0.754 | 0.279 | 0.118 | 0.121 | 0.467 | 0.637 | 0.388 | 0.417 | 0.454 | 0.927 |
| MDA SWS | 0.289 | 0.431 | 0.299 | 0.535 | 0.171 | 0.611 | 0.425 | 0.703 | 0.222 | 0.004 |
